# Case report: A case of tibial tuberous osteochondroma complicated with gouty calculus

**DOI:** 10.3389/fonc.2025.1431601

**Published:** 2025-05-12

**Authors:** Qiang Zhang, Hongjiang Fu, Guomin Ye, Bin Deng, Qun Gao, Ya Chen

**Affiliations:** Department of Medical Imaging, Beijing Jishuitan Hospital Guizhou Hospital, Guiyang, China

**Keywords:** osteochondroma, tibial tuberosity, gouty calculus, imaging diagnosis, surgical resection

## Abstract

Osteochondroma (OC) mainly occurs in the metaphysis of long bones in children and adolescents, and it is extremely rare to occur in the tibial tuberosity and usually has no clinical symptoms. Here, we report a rare case of osteochondroma of the tibial tuberosity combined with gouty stones in a 44-year-old male patient who was found to have a mass on the left upper tibia for 20 years that had not been taken seriously or treated, and the patient was admitted to the hospital after the mass developed painful symptoms. In this case, X-ray examination clearly showed a lesion, which manifested as a limited bony protuberance with a wide base attached to the tibial tuberosity, and the imaging diagnosis was osteochondroma of the tibial tuberosity. The patient underwent surgical resection, and the pathologic results suggested that osteochondroma of the tibial tuberosity was combined with gouty stones. The patient’s symptoms improved significantly after the surgery, and no obvious discomfort or dysfunction has been found in the follow-up so far.

## Introduction

The tibial tubercle is a bony protrusion anterior to the upper end of the tibia, where the quadriceps tendon attaches under the patella. This anatomical structure is not a favored site for osteochondroma or gouty stones, and the occurrence of both of these lesions creates great difficulty for clinicians in making a definitive diagnosis. This similar case has rarely been reported in the literature. We retrospectively analyzed the clinical and imaging data of a patient with osteochondroma of the tibial tuberosity combined with gouty stone to explore the characteristics of its X-ray examination imaging manifestations, with a view to improving the accuracy of the diagnosis of osteochondroma of the tibial tuberosity combined with gouty stone and to provide strong evidence for clinical diagnosis and treatment.

## Case presentation

**Figure 1:**
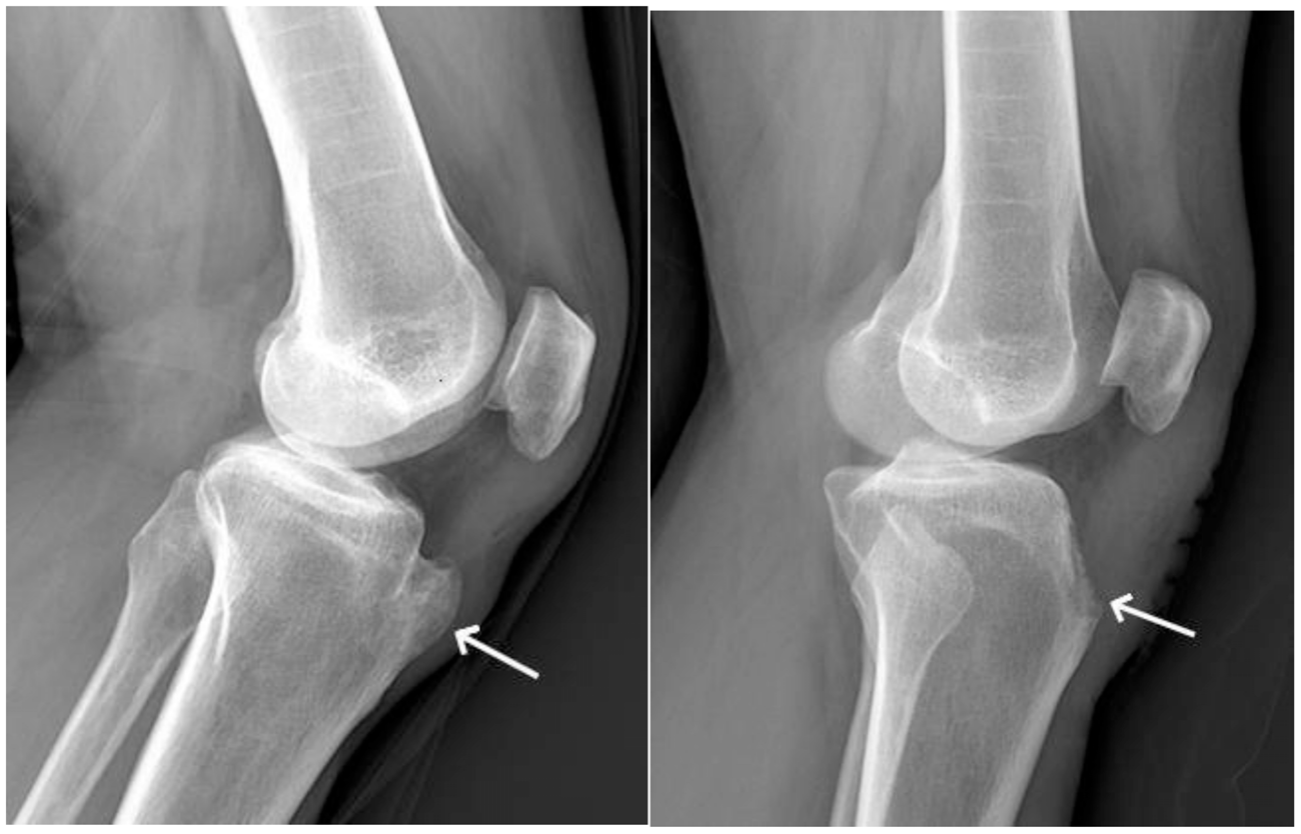
Preoperative radiographs of osteochondroma of the tibial tuberosity combined with gouty calculus (white arrows).

A 44-year-old male patient with no obvious causative factors touched the left upper tibial mass for 20 years, with a size of approximately 3.5 cm × 3.2 cm, but did not pay attention to it. Four years ago, due to gouty arthritis, the patient presented at a local hospital for a knee X-ray examination, which suggested that left tibial tuberosity osteochondroma had not been paid attention to and treated. The patient felt pain in the upper left tibial mass after walking 1 month ago, with no significant change in the size of the mass, redness, and swelling of the left knee joint, which was relieved by rest, and pain in the first metatarsophalangeal joint of both feet. After the onset of the disease, the patient had no symptoms such as low-grade fever, night sweats, fatigue, morning stiffness, joint deformity, and nocturnal rest pain. The patient’s pain symptoms worsened, and he was admitted to the outpatient department for “left tibial osteochondroma”. Physical examination showed that the skin of the left anterior tibial leg was intact, without obvious redness, swelling, or ulceration, and there were no venous varicose veins on the skin surface. Palpation of the anterior margin of the left upper tibial bone revealed a localized bulge on the upper tibial bone of approximately 3.5 × 3.5 cm in size, with a hard texture, clear boundary, and no obvious feeling of fluctuation of the skin; there was a slight sensation of pain on palpation and pressing of the bulging area. Laboratory tests suggested uric acid of 686 μmol/L and blood sedimentation of 60 mm/h, and the rest of the laboratory tests did not show any significant abnormality. Knee X-ray (**Figure 1**) showed a limited bony protrusion anterior to the upper end of the left tibia, with a wide base connected to the tibia, measuring approximately 3.5 cm × 3.4 cm, with no obvious swelling of the surrounding soft tissues, and the knee joint was visible. The imaging diagnosis of osteochondroma of the left tibial tuberosity was highly probable.

**Figure 2:**
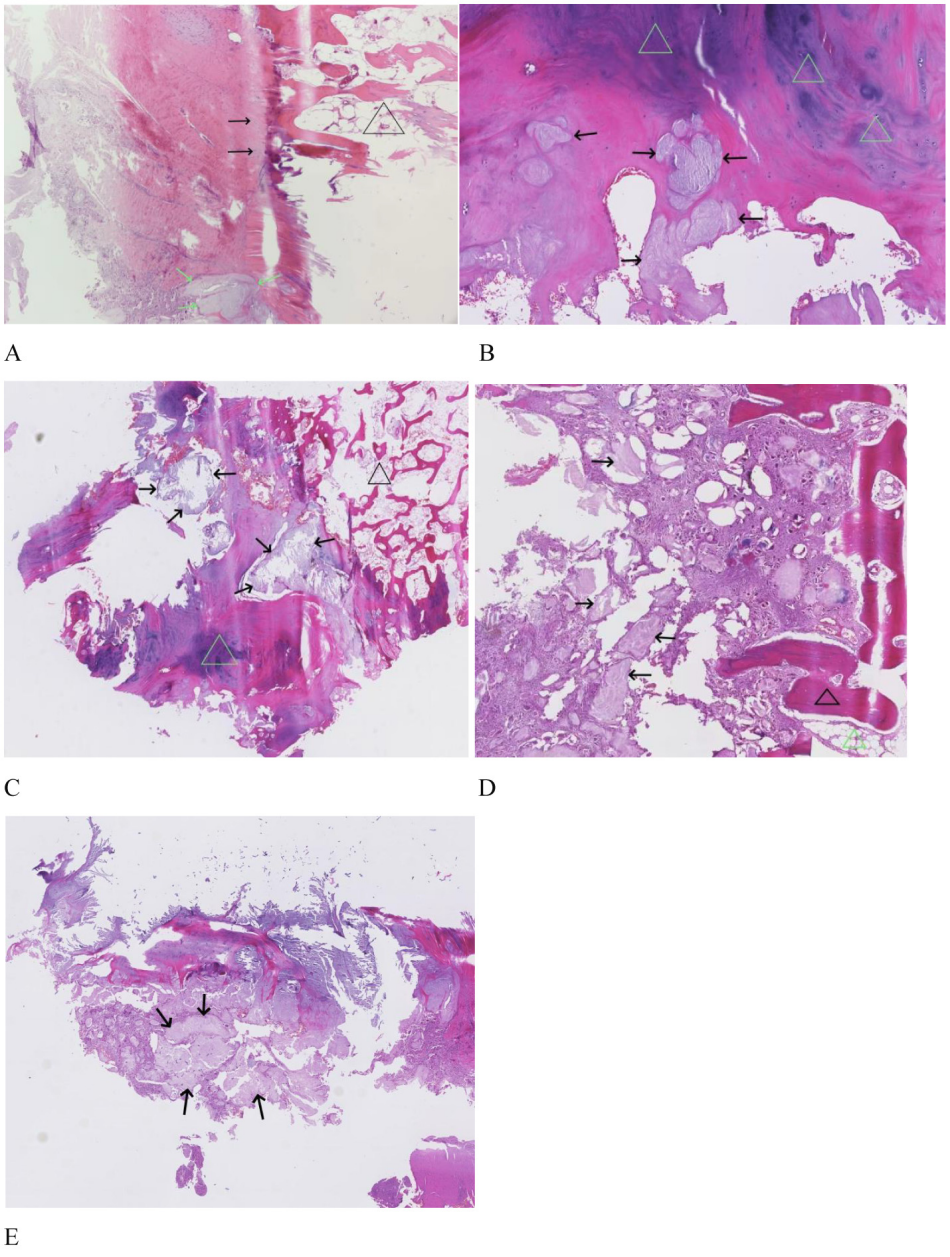
Postoperative radiographs of osteochondroma of the tibial tuberosity combined with gouty calculus (white arrows). FigurePathologic findings of osteochondroma of the tibial tuberosity combined with gouty calculus. **(A)** Black triangles indicate bone marrow, black arrows indicate the cartilage cap, and green arrows indicate urate crystals within the fibrous layer (H&E staining, ×100). **(B)** Green triangles indicate the cartilage cap, and black arrows indicate urate crystals (H&E staining, ×100). **(C)** Black triangles indicate bone marrow, green triangles indicate cartilage cap, and black arrows indicate urate crystals (H&E staining, ×5). **(D)** Black triangles indicate bone trabeculae, green triangles indicate bone marrow, and black arrows indicate urate crystals (H&E staining, ×5). **(E)** Black arrows indicate urate crystals (H&E staining ×5).

In order to clarify the diagnosis and relieve the patient’s pain symptoms, the patient underwent left tibial tubercle resection. During the operation, a curved incision was made on the left tibial tubercle, the length of which was approximately 4 cm. The skin, subcutaneous tissue, and anterior tibial fascia were incised sequentially. A longitudinal incision was made on the patellar tendon and the cartilaginous cap, which revealed osteochondroma. The base of the tubercle was seen to be wider, and it was connected with the tibial tubercle. The tubercle was resected using a bone cutter along the base, the cartilaginous cap was resected, the residual tuberculous bone was secured using bone forceps the probe and resection were thorough, and the incision was localized by electrocautery using an electric knife for high-temperature inactivation. The incision was rinsed with physiological saline, the patellar ligament was repaired, the incision was closed by sewing it from the inside out, and intra-operative specimens were sent for pathologic examinations. Light microscopy showed fibrous tissue membrane, cartilage cap, and loose bone trabeculae with bone marrow hematopoietic components between the trabeculae. Multiple clusters of urate crystals were seen to be deposited, disrupted the cartilage cap and fibrous tissue membrane, and were surrounded by a focus of inflammatory cells and multinucleated giant cell reaction (**Figures 2A–E**). The final diagnosis of pathology was osteochondroma of the tibial tuberosity combined with gouty stone. Postoperative knee radiograph (**Figure 2**) showed that the bony protrusion at the tibial tuberosity had been excised, and the trauma site was smooth with swelling of the surrounding soft tissues.

## Discussion

Osteochondroma (OC) is more common in children and adolescents, being the most common in male individuals. The epiphyseal end of the long shaft is the most common, and it is extremely rare to occur in tibial nodules (1). Patients generally have no symptoms, and the tumor appears as a slowly growing painless mass. When this tumor is large, it can compress surrounding nerves and blood vessels, causing pain and movement disorders (2, 3). X-ray manifestations are as follows: the tumor mostly starts from the metaphysis, grows away from the joint, and migrates to the diaphysis with the growth of the bone. 1) The bony base is the bony superfluous organism that protrudes from the cortex of the parent bone, the one that occurs in the long tubular bone grows away from the articular surface or perpendicular to the diaphysis, which may be widely base or tibial; it continues with the parent bone, the outside is the cortex, and the inside is the cancellous material of the bone. 2) The cartilage cap does not show up on X-ray when it is not calcified, and when calcified, it can be punctiform, annular, semi-annular, and arcuate. Knowledge of X-ray manifestations of OC plays an important role in the pathologic diagnosis of the disease. Osteochondroma pathologically consists of three parts: the body of the tumor with a bony base, the cap composed of hyaline cartilage, and the peritoneum composed of fibrous tissues (4). Surgical resection is the main treatment for OC (5–7).

Gout is a crystal-related joint disease caused by the deposition of monosodium urate (MSU) in joints. It is directly related to hyperuricemia caused by purine metabolism disorders and/or decreased uric acid excretion, characterized by an increase in uric acid in body fluids and blood, and the deposition of urate in various interstitial tissues, leading to inflammatory reactions. The diagnosis of gout can confirmed by fasting blood uric acid >420 μmol/L on two occasions on non-simultaneous days. In patients with gout, urate deposits in the ligaments, bursae, tendon sheaths, and subcutaneous tissues around the joints form gouty nodules (8–10). The bone joints are most likely to be involved, with the first metatarsophalangeal joint being the most common. More than 90% of the patients are male. The edges of the bone-damaging defective areas can be seen to have warped and protruding borders, which are located right on top of the gouty nodules as their characteristics. However, the occurrence of gouty stones in the tibial tuberosity is very rare. Some scholars believe that gouty nodules initially form in the inferior subpatellar bursa of the patellar ligament of the knee due to the movement of the knee joints caused by the gouty nodule upward infiltration breakthrough of the patellar ligament to the subcutaneous tissues and down along the patellar ligament protruding into the tibial tuberosity as the main cause (11).

At the same time, the tibial tuberosity is not a favorable site for OC, and an occurrence in this site needs to be distinguished from Osgood–Schlatter’s disease (12–14), where gouty stones occurring here are easily confused and masked. In this case, OC and gouty stones occurred simultaneously in the tibial tuberosity, which is extremely rare clinically. The gout did not have an acute attack when the patient was admitted to the hospital, so the patient and the doctor easily ignored the history of gout, which caused great difficulties in clinical diagnosis. This led to difficulty in correctly diagnosing the preoperative period, and it was also easy to misdiagnose this as other diseases such as osteochondritis of the tibial tuberosity, hypertrophy of patellar ligament, and fragmentation of the tibial tuberosity (15). After the operation, we followed up with our patient, and the patient recovered well, and no obvious discomfort or dysfunction was found (16).

## Conclusion

In conclusion, the simultaneous occurrence of osteochondroma and gouty stones in the tibial tuberosity is prone to clinical misdiagnosis and underdiagnosis, while giving clinicians warnings and reminders. In the clinic, for patients with osteochondroma diagnosed in the tibial tuberosity, we should closely combine clinical and laboratory examinations if the patient has been diagnosed with gouty arthritis. Even though there is no abnormality of blood uric acid at the time of the current visit, it is still necessary to suspect that osteochondroma is combined with the possibility of gouty tuberosity, and the patient should be closely tracked after the operation to observe the patient’s recovery. At the same time, in the process of imaging diagnosis, attention should be paid to the combination of multiple imaging techniques (17–20), such as CT, MRI, and dual-energy CT examination, in order to make the most correct imaging diagnosis, which was finally diagnosed through pathologic examination.

## Data Availability

The original contributions presented in the study are included in the article/supplementary material. Further inquiries can be directed to the corresponding author.
